# Supplementary material for: Thyroid Stimulating Hormone Levels Are Related to Fatty Liver Indices Independently of Free Thyroxine: A Cross-Sectional Study
Source: J Clin Med. 2025 Mar 31;14(7):2401. doi: 10.3390/jcm14072401 (PMC11990015; doi:10.3390/jcm14072401)
Supplement: Supplementary file 1 [file jcm-14-02401-s001.zip › jcm-3540666-supplementary.pdf]

Supplemental Table S1. Association of TSH levels with steatotic liver indices in our population stratified by sex.

	**Women**		**Men**	
	**β**	***p* value**	**β**	***p* value**
**FLI**	3.22	<0.001	1.51	0.02
**HSI**	0.62	<0.001	0.45	0.01

β values are linear regression coefficients associated with one-point increase in TSH. Models are adjusted for age, fT4 levels, and smoking status. Abbreviations: FLI, fatty liver index; HSI, hepatic steatosis index.

Supplemental Table S2. Association of TSH levels with FIB-4 index in our population.

	**Total N = 2152**	
	**β**	***p* value**
**FIB-4**	0.009	0.26

β values are linear regression coefficients associated with one-point increase in TSH. Models are adjusted for age, sex, fT4 levels, and smoking status.

Supplemental Table S3. Association of TSH levels with FIB-4 index in our population stratified by sex.

	**Women N = 1504**		**Men N = 648**	
	**β**	***p* Value**	**β**	***p* Value**
**FIB-4**	0.011	0.19	0.02	0.24

β values are linear regression coefficients associated with one-point increase in TSH. Models are adjusted for age, fT4 levels, and smoking status.

Supplemental Table S4. Association of TSH levels with insulin resistance in our population stratified by sex.

	**Women**		**Men**	
	**β**	***p* Value**	**β**	***p* Value**
**HOMA-IR**	0.05	0.078	-0.10	0.055

β values are linear regression coefficients associated with one-point increase in TSH. Models are adjusted for age, fT4 levels, and smoking status. Abbreviations: HOMA-IR, Homeostatic Model Assessment for Insulin Resistance.

Supplemental Table S5. Association of TSH levels with insulin resistance in our population stratified by BMI category.

	**Normal weight**		**Overweight and obesity**	
	**β**	***p* Value**	**β**	***p* Value**
**HOMA-IR**	0.241	0.57	-0.07	0.06

β values are linear regression coefficients associated with one-point increase in TSH. Models are adjusted for fT4 levels, age, sex, BMI, and smoking status. Abbreviations: HOMA-IR, Homeostatic Model Assessment for Insulin Resistance.
